# Massive malignant solitary fibrous tumor arising from the bladder serosa: a case report

**DOI:** 10.1186/s13256-014-0505-4

**Published:** 2015-03-01

**Authors:** Jordan Dozier, Zena Jameel, Donald A McCain, Patrice Hassoun, Zubin M Bamboat

**Affiliations:** 1 Department of Surgery, Division of Surgical Oncology, Hackensack University Medical Center, 20 Prospect Avenue, Hackensack, NJ 07601 USA; 2 Department of Pathology, Hackensack University Medical Center, 30 Prospect Avenue, Hackensack, NJ 07601A USA

**Keywords:** Solitary fibrous tumor, Mesenchymal neoplasm, High grade sarcoma

## Abstract

**Introduction:**

Solitary fibrous tumors are rare neoplasms of mesenchymal origin. They are often of low malignant potential and rarely metastasize. While they frequently arise from the pleura, they can occur at any soft tissue site in the body. We present a case of a large (28 × 21cm) malignant solitary fibrous tumor arising from the bladder serosa. In addition, the clinicopathologic features, differential diagnosis, cytogenetics and management of this rare disease are discussed, along with a review of the existing literature on this topic.

**Case presentation:**

An otherwise healthy 41-year-old Caucasian man presented with weight loss and progressive abdominal bloating. A subsequent computed tomography scan of his chest, abdomen and pelvis revealed a 26.8 × 21cm intra-abdominal mass occupying most of his abdominal cavity. The inferior vena cava was compressed, and the mass extended inferiorly to his upper pelvis abutting the superior dome of his bladder. He underwent operative resection and the resected mass measured 28 × 21 × 18cm and weighed 4.8kg. The cut surface revealed a gray-white mass with an ill-defined whorled-like pattern, with randomly assorted tan fleshy nodules. A histologic evaluation revealed variable, alternating hypercellular and hypocellular areas, with areas of necrosis. The tumor cells varied from spindle to epithelioid within a hyalinized stroma. In the hypercellular areas, the tumor cells showed moderate atypia with high mitotic activity. The histological features combined with immunophenotyping were suggestive of a malignant solitary fibrous tumor that grossly appeared to be growing from the bladder serosa, specifically the intraperitoneal superior dome of the bladder. Our patient is currently eight months post-surgery without evidence of recurrence.

**Conclusions:**

Extrapleural occurrences of solitary fibrosis tumors are being increasingly observed. Malignant solitary fibrosis tumors of the urinary bladder, however, are very rare. As there are no pathognomonic features of malignancy, surgical resection is often both diagnostic and therapeutic, as was the case in our report.

## Introduction

In 1942 Stout and Murray first described and defined the hemangiopericytoma (HPC). They described a vascular tumor made of contractile spindle cells surrounding the capillaries [1]. A decade earlier, the first description of a solitary fibrous tumor (SFT) was made by Klemperer and Rabin, who observed a mesothelial tumor arising from the pleura [2]. Since these early observations, two important conclusions have been elucidated. First, HPC was shown to be a characteristic histopathologic pattern rather than being a specific clinicopathologic entity in and of itself. This branching stromal vascular pattern with a ‘staghorn’ configuration is shared by various tumors, including the SFT [3]. Secondly, SFTs have been reported in numerous extrapleural sites, thus supporting the tumor’s undifferentiated mesenchymal origin (with fibroblastic or myofibroblastic features) [4].

In fact, extrapleural SFTs are now more commonly found than intrathoracic SFTs [5]. Specifically, extra-pleural SFT has been described within the head and neck region, central nervous system solid organs, the retroperitoneum, pelvis and the genitourinary tract [6,7]. SFTs are usually benign, and although cases of malignant extrapleural SFT have been described, they are extremely rare. As of 2013, there have been 17 described cases of SFT arising from the urinary bladder, only two of which showed malignant characteristics [7]. We report a case of the largest documented malignant SFT involving the urinary bladder.

## Case presentation

**Figure 1:**
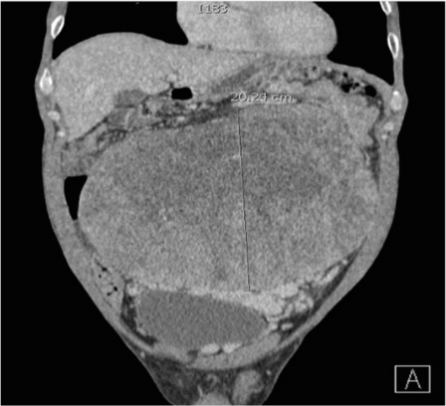
**Coronal section computed tomography scan with intravenous contrast showing a large mass abutting the urinary bladder with engorged pelvic veins.**

A 41-year-old otherwise healthy male presented to the emergency department at Hakensack University Medical Center, NJ with abdominal pain and abdominal fullness that had progressively worsened over the course of the last year. He also reported constipation, urinary frequency, dyspnea on exertion and a 25-pound weight loss. A subsequent computed tomography (CT) scan of his chest, abdomen and pelvis revealed a 26.8 × 21cm intra-abdominal mass occupying most of his abdominal cavity (Figure 1). The inferior vena cava was compressed, and the mass extended inferiorly to the upper pelvis, abutting the superior dome of his bladder. There was no evidence of metastatic disease in his chest. Given the size of the mass and his worsening abdominal pain, the decision was made for him to undergo surgical resection.

The abdomen was entered through a midline incision. The liver and peritoneal surfaces were uninvolved. The mass did not appear to be arising from the retroperitoneum and was noted to be quite mobile, except in the pelvis where it was tethered to the superior dome of the bladder. The mass was freed from the lateral pelvic side walls and a partial cystectomy was performed *en bloc* to remove the specimen. Numerous engorged pelvic veins were encountered around the inferior aspect of the mass and the bladder dome. These were appropriately tied off and stapled using a 45mm vascular load stapler (Covidien, Mansfield, MA, USA). The midline laparotomy was closed in routine fashion.

**Figure 2:**
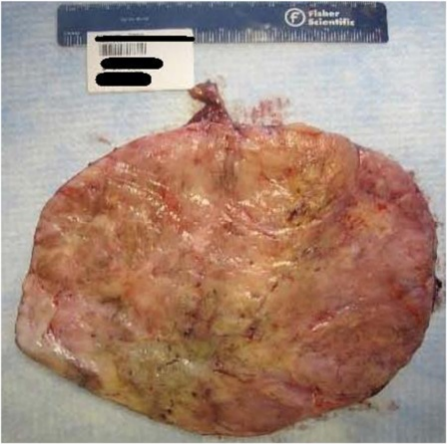
**Bisected gross specimen revealing fleshy, tan nodules.**

**Figure 3:**
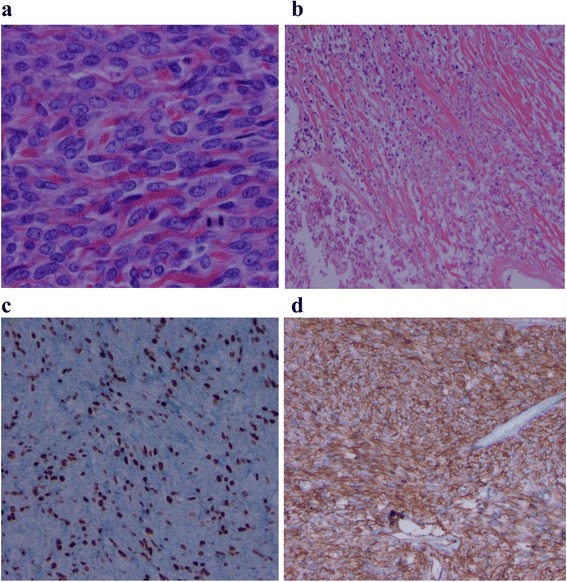
**Histopathology of resected solitary fibrous tumor. a.** Hematoxylin and eosin stain of tumor showing hypercellularity with moderate atypia. **b.** Hematoxylin and eosin stain showing spindle cells within hyalinized stroma in the tumor. **c.** Ki67 proliferation index (>10 mitoses per 10 high power fields, positive cells stained brown). **d.** Diffuse CD34 positivity within the tumor.

On gross inspection, the mass measured 28 × 21 × 18cm and weighed 4.8kg. The cut surface revealed a gray-white mass with an ill-defined whorled-like pattern with randomly assorted tan fleshy nodules (Figure 2). A histologic evaluation revealed a microscopic margin negative resection (R0) with variable, alternating hypercellular and hypocellular areas, with areas of necrosis. The tumor cells varied from spindle to epithelioid within a hyalinized stroma (Figure 3a-b). In the hypercellular areas, the tumor cells showed moderate atypia with high mitotic activity (focally over 10 mitoses per 10 high power fields) and Ki67 positivity of 15% (Figure 3c). His immunohistochemistry analysis showed that the tumor cells were positive for vimentin, CD34 (Figure 3d), BCL-2 and beta-catenin, and negative for pan Cytokeratin, p63, Calretinin, SMA, desmin, S100, CD-31, CD-117, DOG1, EMA, STAT6, GRIA2 and WT-1. His p53 immuno-histochemical stain was weakly positive, with 30 to 40% positivity. His cytogenetics analysis revealed no chromosomal rearrangements at loci 12q13 or 18q11.2.

He had an uneventful post-operative course and was discharged from the hospital on post-operative day five. He was seen in the clinic at one, three and eight months following discharge and is recovering well without evidence of recurrence.

## Discussion

Extrapleural SFTs are most commonly diagnosed between the fifth and seventh decades of life. They are typically slow growing and asymptomatic. Symptomatic tumors are typically secondary to the locally invasive nature of the tumor and compression and/or impingement of nearby structures. Common symptoms seen with intra-abdominal and pelvic SFTs include pain, palpable mass, abdominal distention, urinary retention, hematuria, constipation and bowel obstruction [4]. Overall there is an equal distribution among men and women, however certain SFTs have been shown to have a gender predilection (for example, bladder SFT is 3.5 times more common in males) [7]. A small subset of tumors may cause a paraneoplastic syndrome, the most common of which is hypoglycemia secondary to insulin-like growth factor secreted by the tumor, or so-called Doege-Potter syndrome [4].

Because of the wide variety in clinical presentation, the work-up and diagnosis of SFT can be challenging. Although non-specific, certain imaging characteristics have been observed. A sonography can reveal a hypo- or heterogeneous echogenic mass with well-defined margins. A CT scan typically shows a well-enhanced, circumscribed heterogeneous mass. Prominent vasculature due to mass effect and calcification can be observed in larger lesions, as seen in our case [5]. Regions of hemorrhage and necrosis can also be observed, specifically in cases of malignant SFT. A magnetic resonance imaging scan typically shows variable (low-to-intermediate) signal intensity on T1 and T2 weighted images, dependent on collagenous and fibrous stroma content, vascularity and chronicity of the tumor [8]. Attempts at an image-guided biopsy have been described, but have been largely unsuccessful due to sampling error. As such, surgical resection is often necessary for diagnostic and therapeutic purposes.

Macroscopic examination of solitary fibrous tumors typically reveals a well-circumscribed, tan-colored rubbery mass with a white whorled appearance on cut sections. The mass is often tethered by a pedicle and encapsulated. Most cases of bladder SFT are described as being intravesicular, growing from the submucosa, however there have been isolated cases of pelvic SFT growing from the serosal surface of the bladder, as was seen with our patient. Microscopically, SFT is classically described as having a ‘patternless pattern’, with juxtaposed hypercellular (spindle to ovoid cells) and hypocellular zones (hyalinized collagen) and a prominent branching vasculature [4,7]. Mitoses and microscopic necrosis are rare in SFTs. Because there are numerous other spindle cell lesions that can display a similar cellular architecture (such as gastrointestinal stromal tumor, leiomyoma, leiomyosarcoma, malignant fibrous histiocytoma, benign and malignant nerve sheath tumor and so forth), the immunohistochemical staining plays an important role in the diagnosis, as seen in our case. The tumor cells are often positive for CD34, CD99, vimentin and BCL-2, and negative for CD117 (in gastrointestinal stromal tumors), smooth muscle actin (SMA) and desmin (in smooth muscle tumors) and S-100 protein (in nerve sheath tumors) [6,7]. Recently, genomic studies have revealed a gene fusion that may have diagnostic and therapeutic implications for patients with SFT. The *NAB2* gene which indirectly represses transforming growth factor (TGF) - β, and the *STAT6* gene which is a transcriptional factor that modulates signaling through interleukin-4 and interleukin-13, have both been described as oncogenic. A recent study of 53 patients undergoing whole exome tumor sequencing revealed that when fused, these two genes (located on chromosome 12) represent a distinct molecular feature in SFT. This characteristic fusion transcript occurred in 55% of tumors, creating the opportunity to develop drugs that specifically target the fusion gene product. Interestingly, a cytogenetic analysis did not reveal the *NAB2*-*STAT6* fusion in our patient [9]. Moreover, an immunohistochemistry analysis for *STAT6* and *GRIA2* was also negative. An analysis of 44 SFTs by Mohajeri *et al*. revealed that in addition to the *NAB2*-*STAT6* fusion transcript, GRIA2 was the top up-regulated protein found by immunohistochemistry using tissue microarrays [10].

Malignant criteria for SFTs include large tumor size (>10cm), hypercellularity, nuclear atypia, tumor necrosis, more than four mitoses per 10 high power fields and infiltrative margins [5]. However, it is important to note that malignant histologic features are not always an indicator of aggressive tumor behavior, as benign tumors can act locally aggressive and recur, while alternatively malignant tumors can proceed with an indolent course [4,7]. Nonetheless, with respect to benign SFTs, malignant SFTs do account for higher rates of local recurrence (63 versus 8%) and metastasize to distant soft tissue sites and the lungs [11]. With respect to bladder SFT, most reported cases have pursued a benign course, even in the presence of malignant histologic features [7].

Because of the variable clinical behavior and unpredictable nature of SFTs, surgery is considered the treatment of choice. Furthermore, long-term follow-up is strongly recommended for all cases of SFT, although no specific surveillance strategy has proved to be superior. Surgical resectability is the most important prognostic factor, and the five-year survival rate is high, with some authors claiming close to 100% with complete surgical excision (R0 resection) [5]. For unresectable disease, chemotherapy and radiation therapy have been described as having variable success. In one retrospective study of 21 patients with late-stage SFT, 16 of the 18 patients who received first line chemotherapy (with doxorubicin, gemcitabine or paclitaxel) had no disease progression for a median of 4.6 months [12]. Prospective randomized studies are needed to substantiate these results. Going forward, anti-angiogenic therapy (such as bevacizumab, a monoclonal antibody against vascular endothelial growth factor) has shown promising early results in the treatment of patients with advanced unresectable tumors [12].

## Conclusions

We report the case of a 41-year-old man who presented with the largest documented case of malignant SFT arising from the urinary bladder. While there are characteristic findings suggestive of SFT, no gold standard diagnostic test currently exists. As such, surgical excision is both therapeutic and diagnostic when combined with subsequent histological, immunohistochemical and genomic testing.

## Consent

Written informed consent was obtained from the patient for publication of this Case report and any accompanying images. A copy of the written consent is available for review by the Editor-in-Chief of this journal.

## Abbreviations

CT: Computed tomography
IHC: Immunohistochemistry
SFT: Solitary fibrous tumor. SMA-Smooth Muscle Antibody
EMA: Anti-endomysial Antibodies
GRIA2: Glutamate Receptor 2
WT-1: Wilms Tumor Protein
DOG1: Discovered on GIST1
BCL2: B-Cell Lympoma 2
